# Hypermethylation of the *ALOX12* and *CBS* promoters in osteoporosis: Potential biomarkers for early diagnosis

**DOI:** 10.1016/j.gendis.2023.03.005

**Published:** 2023-04-10

**Authors:** Shang Guo, Yue Wu, Weekai Chia, Peichun Hsu, Hongwei Wang, Yunliang Wang, Biao Zhong

**Affiliations:** a Department of Orthopedic Surgery, Shanghai Sixth People's Hospital Affiliated to Shanghai Jiao Tong University School of Medicine, Shanghai 200023, China; b Institute of Translational Medicine, Shanghai General Hospital, Shanghai Jiao Tong University School of Medicine (SJTU-SM), Shanghai 200025, China; c The University of Chicago, Department of Medicine, Chicago, IL 60637, USA; d Department of Neurology, The Second Affiliated Hospital of Zhengzhou University, Zhengzhou, Henan 450014, China

As a bone-confined chronic degenerative disorder, the progression of osteoporosis is characterized by abnormal crosstalk between osteoblasts and osteoclasts, leading to an imbalance of bone remodeling in adults.^1^ Several studies reported that arachidonic acid ester 12 lipoxygenase (ALOX12) acts as a regulator in bone genesis by participating in the activation of the peroxisome proliferator-activated receptor γ (PPARG) pathway through its reaction product.^2^ A positive correlation between *ALOX12* gene polymorphism and bone mineral density (BMD) has also been verified,^3^ indicating that serum Se deficiency was accompanied by some *ALOX12* variation, contributing to the peak BMD and the development of osteoporosis. Another multidomain enzyme involved in DNA methylation, cystathionine beta-synthase (CBS), regulates the conversion of homocysteine into glutathione. Mutations in CBS lead to the production of more sulfur end-products from the methylation cycle. The newborn CBS-knockout (KO) mice who received treatment with recombinant poly ethylene glycol human truncated CBS (PEG-CBS) were rescued from osteoporosis-like symptoms.^4^ In patients with osteoporosis, the expression of *CBS* was found to be down-regulated in femur tissues, leading to a lower BMD.^5^ These findings support the idea that DNA methylation-related enzymes, including ALOX12 and CBS, are important for the occurrence of osteoporosis. However, the specificities in these 2 genes of osteoporosis human samples are largely unclear. Thus, we aimed to explore the impact of the DNA methylation level on select candidate genes in osteoporosis. These results will be of great help in optimizing treatments and for the early diagnosis of osteoporosis, complying with the principles of precision medicine.

We first performed bisulfite conversion assays on both *ALOX12* (Fig. 1A) and *CBS* (Fig. 1B). The sequencing results for the bisulfite-treated qMSP products are shown on the left part, confirming the complete bisulfite conversion. The right part shows the peaks after capillary electrophoresis, demonstrating that the lengths of the qMSP products were in line with the expectations. By characterizing the target sequence of human *ALOX12* (Fig. S1B) and *CBS* genes (Fig. S1C), the methylation involving CpG islands in the promoter region on the qMSP primer fragments for both genes is highlighted in gray.

**Figure 1:**
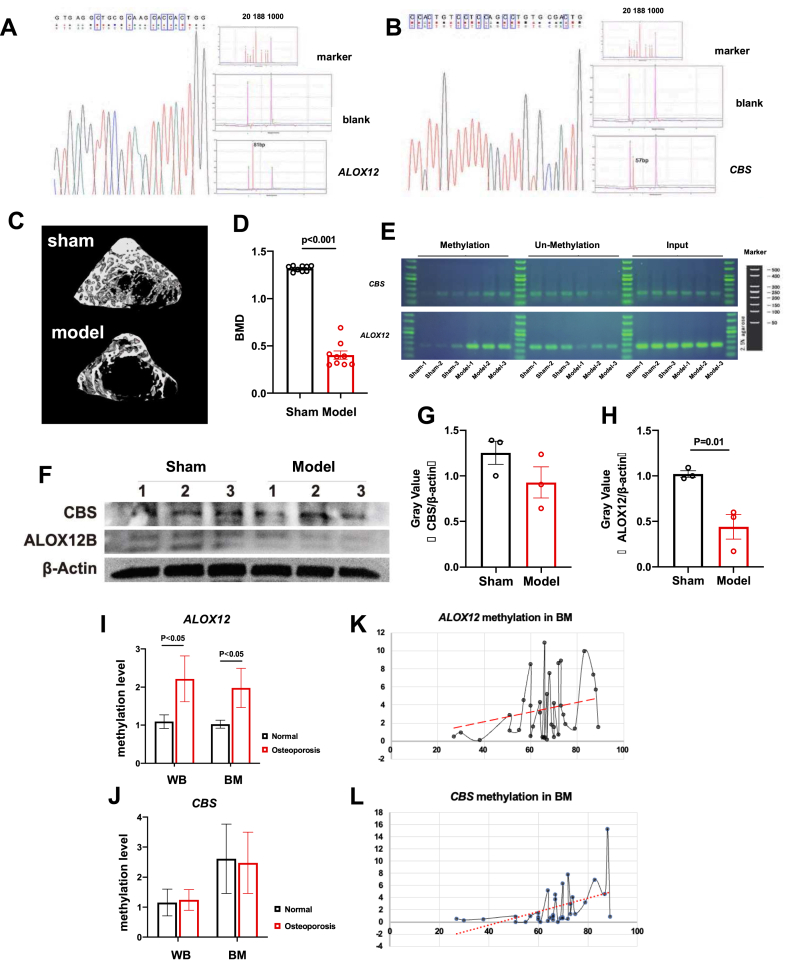
Hypermethylation within the ALOX12 and CBS promoters in a rat model of osteoporosis and human osteoporosis patients. **(A, B)** qMSP results showing the methylation of *ALOX12* (A) and *CBS* (B). **(C)** Representative micro-CT images of rats from the sham and ovariectomy groups. **(D)** Bone mineral density (BMD) analysis of rats in the two groups. **(E)** DNA methylation results for Alox12 and Cbs in the peripheral blood 12 weeks after surgery. **(F)** Western blot analysis of the ALOX12 and CBS expression in the rats. **(G, H)** Gray value statistical analysis of the CBS (G) and ALOX12 (H) expression. **(I, J)** Histograms showing the DNA methylation level of *ALOX12* (I) and *CBS* (J). WB, whole blood. BM, bone marrow. **(K, L)** Correlation chart between age and DNA methylation level in all bone marrow osteoporosis samples.

To investigate the contribution of DNA methylation in *ALOX12* and *CBS* during osteoporosis, we performed ovariectomy to induce osteoporosis-like symptoms in rats (Fig. S2A). Before we performed the surgeries, rats were pre-treated with antibiotics for 1 week and then observed for 12 weeks post-surgery (Fig. S2B). Micro-CT imaging and related analyses showed that the ovariectomized rats had a significantly decreased BMD compared with that in the sham group after 12 weeks (Fig. 1C, D). We further found a notable decrease in the bone volume fraction, trabecular thickness, and the trabecular number of the ovariectomized rats (Fig. S2C–E), demonstrating that our rat model had symptoms consistent with osteoporosis.

We then studied the DNA methylation levels of *ALOX12* and *CBS* in rats. After extracting DNA from whole blood samples of osteoporotic rats, we detected gene methylation based on PCR analyses. The results showed that the model group had higher methylation levels in both the *ALOX12* and *CBS* genes compared with sham controls (Fig. 1E). Moreover, the protein expression of ALOX12 and CBS was also lower in the osteoporosis model group as indicated by Western blotting (Fig. 1F). The expression of ALOX12 was more extensively decreased (as indicated in the gray values) (Fig. 1G, H). Taken together, our experiments in the rat model demonstrate that there is hypermethylation of *ALOX12* and *CBS*, and this correlated with the presence of osteoporosis and with decreased protein expression of these targets.

To validate our above findings, a total of 46 osteoporosis patients with 30 age- and gender-matched normal controls were enrolled in this study as shown in Table 1. Whole blood and bone marrow samples were both collected for testing. We first performed a qMSP experiment to detect the methylation levels of *ALOX12* and *CBS* in the patient samples. Our results showed that the *ALOX12* gene methylation levels in the samples from patients with osteoporosis were significantly higher than those in normal controls (Fig. 1I). This was noted in both whole blood and bone marrow samples. In a further subgroup analysis, the bone marrow samples from males and whole blood samples from females with osteoporosis also had significantly higher *ALOX12* methylation levels than the normal controls (Fig. S3A, B). However, there were no significant differences in the methylation levels of *CBS* between different genders or total samples (Fig. 1J; Fig. S3C, D), indicating the age impactor may play a more important role in integrating with *CBS* hypermethylation. Besides, the variations of *ALOX12* and *CBS* in bone marrow samples with age showed clear positive correlations both in separate samples (Fig. 1K, L) and grouping analysis (Fig. S4A, C).

**Table 1:** Baseline characteristics of the study participants.

	Normal (*n* = 30)	Osteoporosis (*n* = 46)	*P* value
Age (years)	58.33 ± 2.01	63.19 ± 2.03	0.123
Gender (male/female)	14 / 16	13 / 33	
BMD (g/cm^2^)	1.01 ± 0.11	0.75 ± 0.10	<0.0001
WBCs (/μL)	5.00 (2.00, 21.50)	9.00 (4.50, 22.00)	0.178
RBCs (/μL)	13.00 (5.00, 20.00)	14.00 (7.00, 45.00)	0.219
EPIs (/μL)	4.00 (7.00, 15.00)	3.00 (6.00, 13.00)	0.325
Glucose (mmol/L)	5.15 ± 0.57	5.45 (4.90, 5.80)	0.073
UA (μmol/L)	299.15 ± 72.12	268.87 ± 84.60	0.080
Creatinine (μmol/L)	64.48 ± 12.32	61.71 ± 12.03	0.037
Urea (mmol/L)	5.09 ± 1.77	5.64 ± 1.65	0.127
T-BIL (μmol/L)	23.18 ± 8.69	21.25 ± 7.77	0.260
TP (g/L)	66.09 ± 4.47	65.78 ± 5.87	0.790
Albumin (g/L)	39.67 ± 3.37	37.03 ± 3.84	0.105
Ca (mmol/L)	2.19 ± 0.08	2.19 ± 0.10	0.907
CO2 (mmol/L)	26.00 (25.50, 27.00)	25.00 (25.00, 28.00)	0.096
Serum K (mmol/L)	3.77 ± 0.42	3.90 ± 0.48	0.164
Serum Na (mmol/L)	140.91 ± 2.04	142.00 (140.00, 143.00)	0.095
Serum Cl (mmol/L)	101.85 ± 1.91	103.00 (102.00, 104.00)	0.012
ALT (μ/L)	36.12 ± 3.22	40.26 ± 3.11	0.848
AST (μ/L)	27.30 ± 13.21	22.00 (19.00, 28.00)	0.926
γGT (μ/L)	26.68 ± 11.88	23.70 ± 9.84	0.196
ALP (μ/L)	68.38 ± 1.65	72.48 ± 2.01	0.157
LDH (μ/L)	499.00 ± 131.24	548.29 ± 112.29	0.053

An independent-samples *T* test was used.

BMD: bone mineral density; WBC: white blood cell; RBC: red blood cell; EPI: epithelial cell; UA: uric acid; T-BIL: total bilirubin; TP: total protein; ALT: glutamic-pyruvic transaminase; AST: glutamic oxalacetic transaminase; γ-GT: γ-glutamyl transferase; ALP: alkaline phosphatase; LDH: lactate dehydrogenase.

∗Chi-square test was applied; ^a^Nonparametric test was used; ^b^Independent-Samples *T* test was used.

Thus, we confirmed that there is hypermethylation of *ALOX12* in both the rat model of osteoporosis and human patients with osteoporosis, suggesting that it may participate in the development of this disease. It may therefore have the potential as an easy-to-obtain early diagnostic biomarker for further investigation, especially for patients who cannot undergo x-rays or DEXA. It would also be a safe and cheaper alternative to DEXA which has therapeutic potential as well.

## Ethics declaration

The human study was approved by the Ethics Committees of Shanghai Sixth People's Hospital Affiliated to Shanghai Jiao Tong University (Shanghai, China) (No. 2021-YS-093). For age- and sex-matched rats, animals were fed and maintained under specific pathogen-free conditions following the criteria of the National Institutes of Health (Bethesda, MD) Guide for the Care and Use of Laboratory Animals with the approval of the Ethics Committees of Shanghai Sixth People's Hospital Affiliated to Shanghai Jiao Tong University (Shanghai, China) (No. 2022–0271).

## Author contributions

S.G. wrote the main manuscript and acquired the funding; Y.W. contributed to the methodology of experiments needed and data analysis; W.C. and P.H. did the investigation and data curation; W.C. reviewed the writing; H.W. did the validation; Y.W. and B.Z. made the supervision and reviewed and edited the manuscript.

## Conflict of interests

The authors declare no conflict of interests.

## Funding

This work is supported by grants from the Interdisciplinary Program of 10.13039/501100004921Shanghai Jiao Tong University (No. YG2019QNA23).

## References

[1] Marini F., Cianferotti L., Brandi M.L. (2016). Epigenetic mechanisms in bone biology and osteoporosis: can they drive therapeutic choices?. Int J Mol Sci.

[2] Xiao W.J., He J.W., Zhang H. (2011). ALOX12 polymorphisms are associated with fat mass but not peak bone mineral density in Chinese nuclear families. Int J Obes.

[3] Al-E-Ahmad A., Parsian H., Fathi M. (2018). *ALOX12* gene polymorphisms and serum selenium status in elderly osteoporotic patients. Adv Clin Exp Med.

[4] Majtan T., Hůlková H., Park I. (2017). Enzyme replacement prevents neonatal death, liver damage, and osteoporosis in murine homocystinuria. FASEB J.

[5] Hao Y.M., He D.W., Gao Y. (2021). Association of hydrogen sulfide with femoral bone mineral density in osteoporosis patients: a preliminary study. Med Sci Monit.

