# Supplementary material for: Hypermethylation of the ALOX12 and CBS promoters in osteoporosis: Potential biomarkers for early diagnosis
Source: Genes Dis. 2023 Apr 10;11(1):30–3. doi: 10.1016/j.gendis.2023.03.005 (PMC10425782; doi:10.1016/j.gendis.2023.03.005)
Supplement: Multimedia component 1 [file mmc1.docx]

**Supplementary Materials**

**Materials and Methods**

**Human subjects**

Forty-six patients with osteoporosis and 30 healthy matched controls were enrolled after providing written informed consent, and samples were collected in accordance with the diagnostic criteria. The study was approved by the ethics committees of Shanghai Sixth People’s Hospital Affiliated with Shanghai Jiao Tong University (Shanghai, China) (No. 2021-YS-093). The whole blood and bone marrow samples are acquired in every participant. Clinical information including the gender, age, smoking history, history of hypertension, history of diabetes, etc., was obtained from the medical records.

**Animals**

Sprague Dawley rats were purchased from the Shanghai Laboratory Animal Center (Shanghai, China). Age- and sex-matched rats were fed and maintained under specific pathogen-free conditions following the criteria of the National Institutes of Health (Bethesda, MD) Guide for the Care and Use of Laboratory Animals with the approval of the ethics committees of Shanghai Sixth People’s Hospital Affiliated with Shanghai Jiao Tong University (Shanghai, China) (NO 2022-0271).

**Ovariectomy-induced animal model of osteoporosis**

Female Sprague Dawley rats (200‒250 g) were anesthetized as described previously. Briefly, rats were injected intraperitoneally with 10% chloral hydrate at 4-5 μl/g after fur was removed from the abdomen. Rats in the model group rats underwent ovariectomy while 1g fat resection was performed in the rats in the sham group. All rats were kept at 36℃ during the surgery. Samples were collected at the 8th and 12th weeks post- surgery.

**Bisulfite conversion and qMSP**

Genomic DNA extraction and bisulfite conversion were performed using procedures described in previous study ^1^. Briefly, the DNA methylation of the gene promoter was predicted, and the MSP primers for *CBS* and *ALOX12* were designed using the MethPrimer online software (http://www.urogene.org/methprimer/). Then, the methylation status of the *CBS* and *ALOX12* promoters was assessed by MSP analysis.

Briefly, the genomic DNA of rats was extracted using a TIANamp Genomic DNA Kit (TIANGEN biotech, Beijing, China) based on the manufacturer’s instructions. The genomic DNA (500 ng) was treated with sodium bisulfite with an EZ DNA Methylation Gold Kit (Zymo Research Corp., CA, USA). Next, the DNA was converted based on the manufacturer’s instructions. Then, the converted DNA was used for PCR with primers specific for unmethylated or methylated *CBS* and *ALOX12*. Ultimately, the PCR products were observed using 2% agarose gel electrophoresis. The primers used in MSP are listed below.

M-rCBS：

Forward primer (F) 5’-TACGTTAAGTTAGGAAGTTTTACGA-3’

Reverse primer (R) 5’-TATACCCAAAAATCTACACCCTACG-3’

UM-rCBS：

Forward primer (F) 5’-TTATGTTAAGTTAGGAAGTTTTATGA-3’

Reverse primer (R) 5’-TACCCAAAAATCTACACCCTACAAC-3’

M-rALOX12：

Forward primer (F) 5’-TTTTTTTTGAGATTTAAGATTAGGC-3’

Reverse primer (R) 5’-TATACCCAAAAATCTACACCCTACG-3’

UM-rALOX12：

Forward primer (F) 5’-TTATGTTAAGTTAGGAAGTTTTATGA-3’

Reverse primer (R) 5’-TACCCAAAAATCTACACCCTACAAC-3’

**Western blotting**

Rat bone marrow specimens were homogenized and lysed in radio- immunoprecipitation buffer (Beyotime Biotechnology, China) supplemented with protease and phosphatase inhibitor cocktails (ApexBio Technology, USA). Proteins were then separated by 10% SDS-PAGE and transferred onto polyvinylidene difluoride membranes. Anti-ALOX12 (Abways), anti-CBS (Proteintech), and anti‒b-III tubulin (Abcam) antibodies were used as the primary antibodies, followed by incubation with HRP-conjugated secondary antibodies (Proteintech). Signals were then detected with an ECL chemiluminescent substrate kit (Yeasen Biotechnology, China) and imaged with a GE Amersham Imager 600 (GE Healthcare, Amersham, United Kingdom).

**Detection of telomere length**

All samples were used in the detection of telomere length (TL). Each reaction was run using 2x MasterMix (5 μl), primers (0.5 μl per primer), template DNA (1.5 μl), and H_2_O (3 μl). Samples were added to a 384-well plate, where each plate contained 36b4 as a positive control (Thermo Fisher Scientific, Uppsala, Sweden) with ddH_2_O used as the negative control. Real-time reverse transcription PCR (RT-PCR) reactions were run using a LightCycler® 480 (Roche, Basel, Switzerland). The PCR conditions were: an initial denaturation process at 95℃ for 10 min followed by 45 cycles of 95℃ for 15s, and 60℃ for 1 min, and dissociation curves were mapped at 95℃ for 15s, 60℃ for 1 min, 95℃ for 10s, and a final cool-down step at 40℃ for 10s. The telomere repeat copy number (T) and the internal single copy reference gene 36B4 gene copy number (S) were detected. PCR reactions were performed using the standard diluted by multiple ratios and the samples to be tested. The cycle threshold (Ct) value represents the number of cycles required for the fluorescence to cross the set threshold. The standard curve was calculated based on a Ct value of 4 standard dilutions to analyze the copy number of sample DNA. The formula used to calculate telomere length was as follows: telomere length = T/S/92×6. Our procedures for detecting telomere length were performed in accordance with the previous study ^2^.

**Statistical analysis**

The data conforming to a normal distribution are presented as the mean ± SEM, while the data that showed a skewed distribution are presented as the median (interquartile range). All data were analyzed using GraphPad Prism 8 (GraphPad Software, San Diego, CA). Student’s t-test was used for the comparison of 2 groups. Probability values <0.05 were considered significant.

**Supplementary Figure 1.** Gene characteristics of ALOX12 and CBS in osteoporosis.

**A**, Schematic illustration of DNA hypermethylation in osteoporosis. **B, C**, The characteristics of *ALOX12* (**B**) and *CBS* (**C**).

**Supplementary Figure 2.** Construction of ovariectomy osteoporosis rat model.

**A,** Cartoon characterization of the ovarian resection. **B,** Schematic diagram of the ovariectomy-induced osteoporosis rat model and the observation period. **C**, BV/TV percentage analysis between control and model rat groups. **D,** Tb.Th analysis between control and model rat groups. **E**, Tb.N analysis between control and model rat groups.

**Supplementary Figure 3.** Gender-related subgroup analysis of *ALOX12* and *CBS* DNA methylation level.  **(A, B)** DNA methylation level of *ALOX12* in different gender groups of whole blood **(A)** and bone marrow **(B)** samples. **(C, D)** DNA methylation level of *CBS* in different gender groups of whole blood **(C)** and bone marrow **(D)** samples.

**Supplementary Figure 4.** Age-related subgroup analysis of ALOX12 and CBS DNA methylation level in all osteoporosis samples. **(A, B)** DNA methylation level of *ALOX12* in different age groups of bone marrow **(A)** and whole blood **(B)** samples. **(C, D)** DNA methylation level of *CBS* in different gender groups of bone marrow **(C)** and whole blood **(D)** samples.

**References Cited**

1. Martisova A, Holcakova J, Izadi N, et al. DNA Methylation in Solid Tumors: Functions and Methods of Detection. *Int J Mol Sci*. 2021; 22(8):4247.
2. Aubert G, Hills M, Lansdorp PM. Telomere length measurement-caveats and a critical assessment of the available technologies and tools. *Mutat Res*. 2012; 730(1-2):59-67.
